# Supplementary material for: Structural insights into the allosteric inhibition of P2X4 receptors
Source: Nat Commun. 2023 Oct 13;14:6437. doi: 10.1038/s41467-023-42164-y (PMC10575874; doi:10.1038/s41467-023-42164-y)
Supplement: Supplementary file 1 — Supplementary information [file 41467_2023_42164_MOESM1_ESM.pdf]

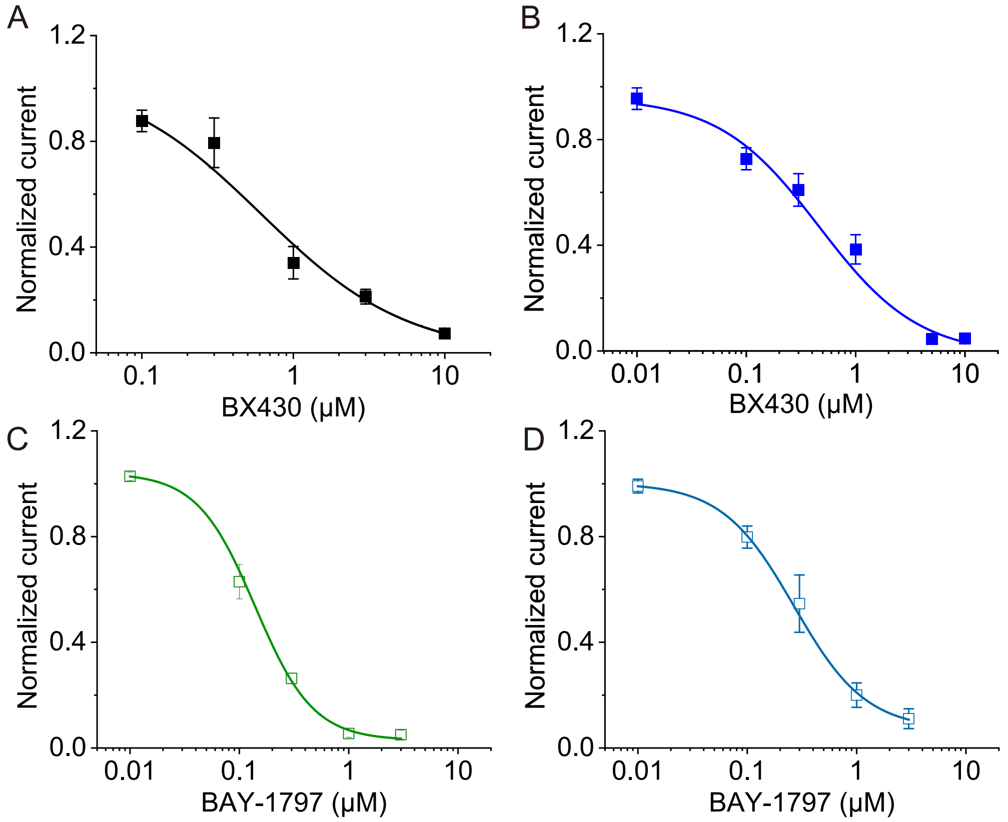

Supplementary Figure 1. Inhibitory effects of BX430 and BAY-1797 on P2X4 receptors.

(A) Concentration–response curves of BX430 on zebrafish P2X4 (1 mM ATP concentration tested). The estimated IC50 value was 0.61 ± 0.66 µM (n=3-4 for each tested BX430 concentration). (B) Concentration–response curves of BX430 on human P2X4 (100 µM ATP tested). The estimated IC50 value was 0.46 ± 0.22 µM (n=7-9 for each tested BX430 concentration). (C) Concentration–response curves of BAY-1797 on zebrafish P2X4 (1 mM ATP tested). The estimated IC50 value was 0.14 ± 0.03 µM (n=3-4 for each tested BAY-1797 concentration). (D) Concentration–response curves of BAY-1797 on human P2X4 (10 µM ATP tested). The estimated IC50 value was 0.24 ± 0.03 µM (n=3-7 for each tested BAY-1797 concentration). The data are presented as the mean ± SEM.

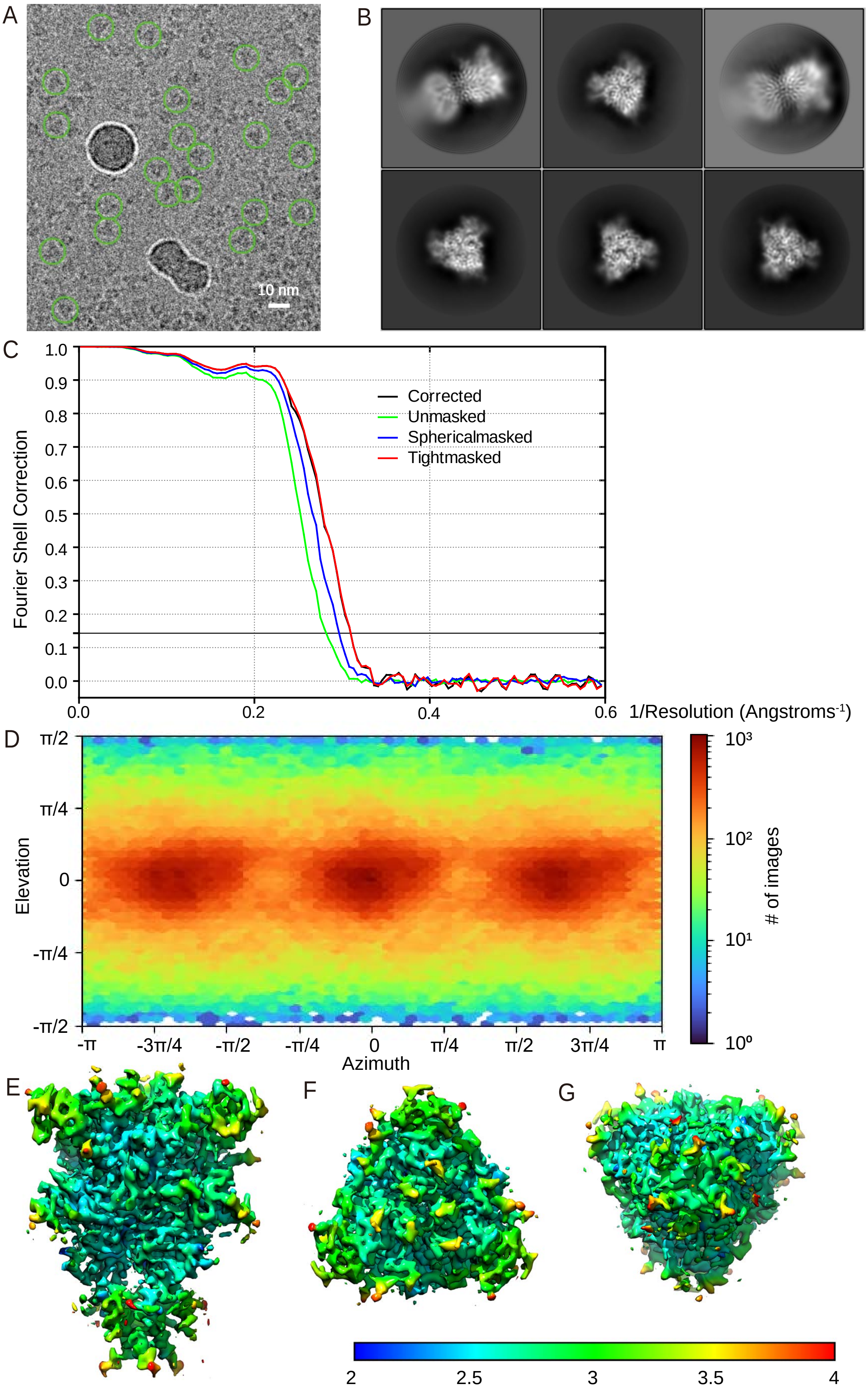

Supplementary Figure 2. Cryo-EM analysis of zebrafish P2X4 in complex with BX430.

(**A**) Representative cryo-EM image of zebrafish P2X4 particles in complex with BX430. (**B**) Representative 2D class averages. (**C**) The gold-standard Fourier shell correlation (FSC) curves for resolution estimation. (**D**) Angular distribution of the particles used for the final map. (**E-G**) Side view (**E**), top view from the extracellular side (**F**), and bottom view from the cytoplasmic side (**G**) of the EM density map, colored according to the local resolution, estimated using CryoSPARC.

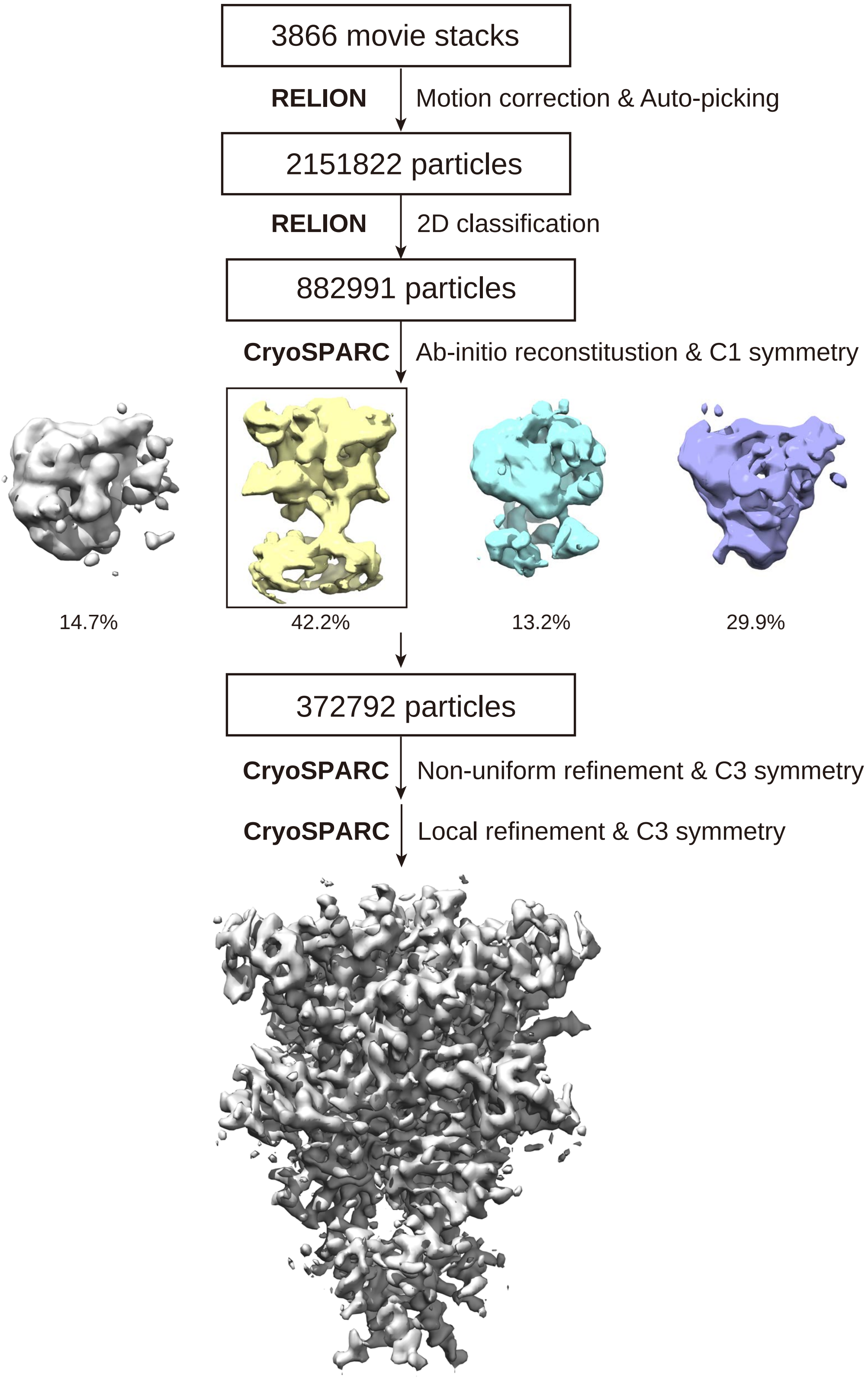

Supplementary Figure 3. Cryo-EM data processing workflow for zebrafish P2X4 in complex with BX430.
All processing steps were performed in RELION and CryoSPARC. Images were generated using UCSF Chimera.

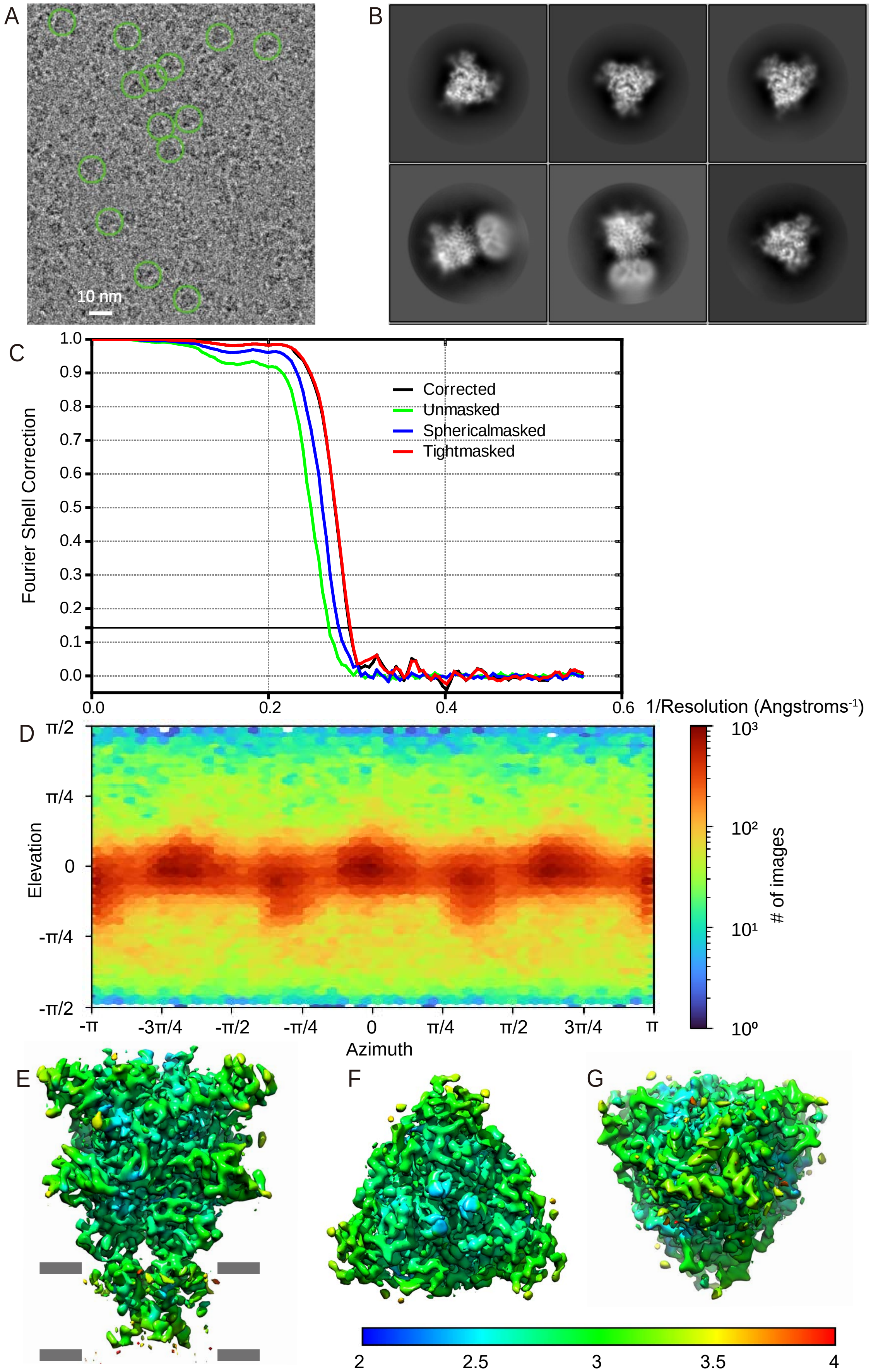

Supplementary Figure 4. Cryo-EM analysis of zebrafish P2X4 in complex with BAY-1797.

(**A**) Representative cryo-EM image of zebrafish P2X4 particles in complex with BAY-1797. (**B**) Representative 2D class averages. (**C**) The gold-standard Fourier shell correlation (FSC) curves for resolution estimation. (**D**) Angular distribution of the particles used for the final map. (**E-G**) Side view (**E**), top view from the extracellular side (**F**), and bottom view from the cytoplasmic side (**G**) of the EM density map, colored according to the local resolution, estimated using CryoSPARC.

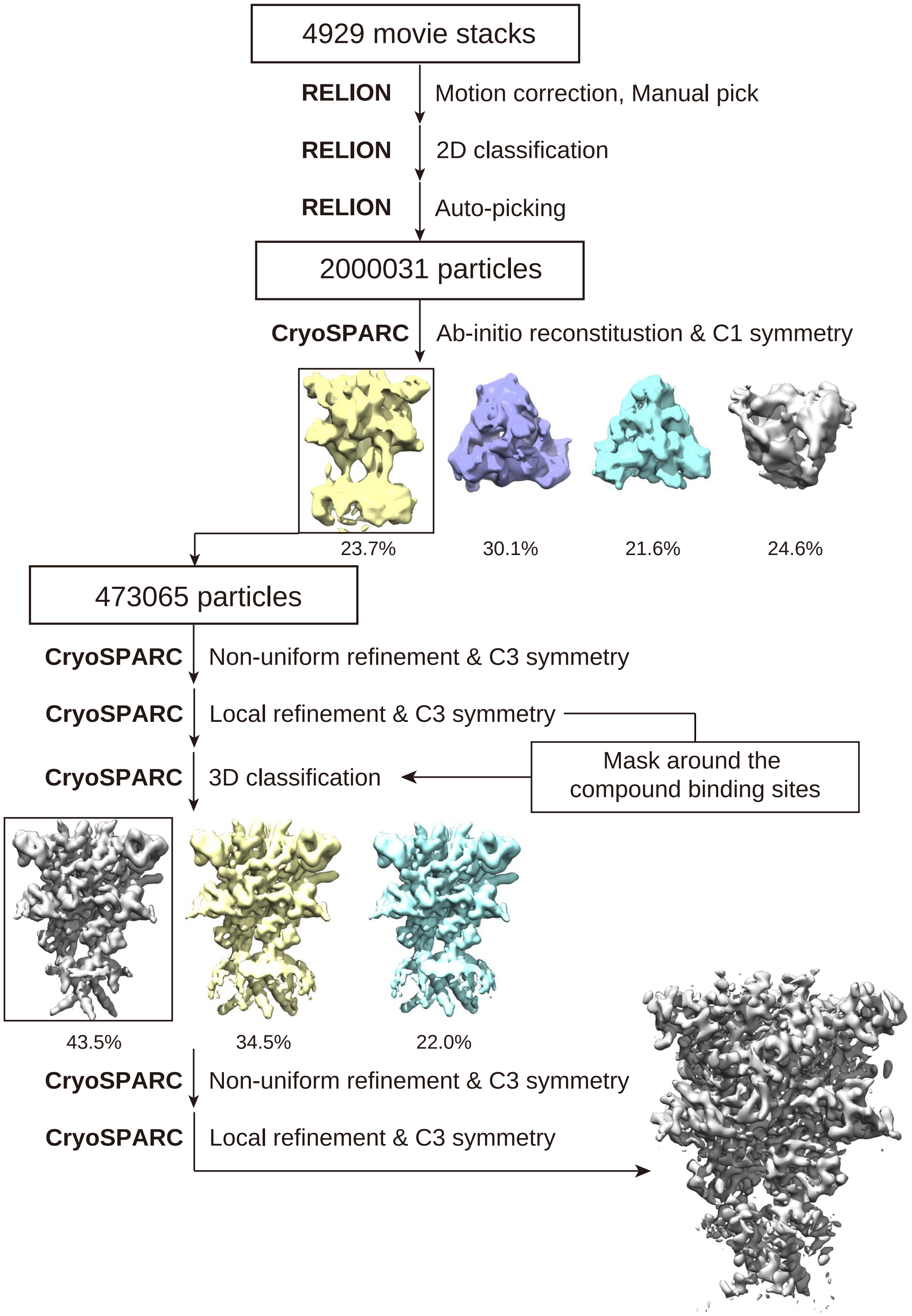

Supplementary Figure 5. Cryo-EM data processing workflow for zebrafish P2X4 in complex with BAY-1797.
All processing steps were performed in RELION and CryoSPARC. The mask around the compound binding sites was generated using UCSF Chimera. Images were generated using UCSF Chimera.

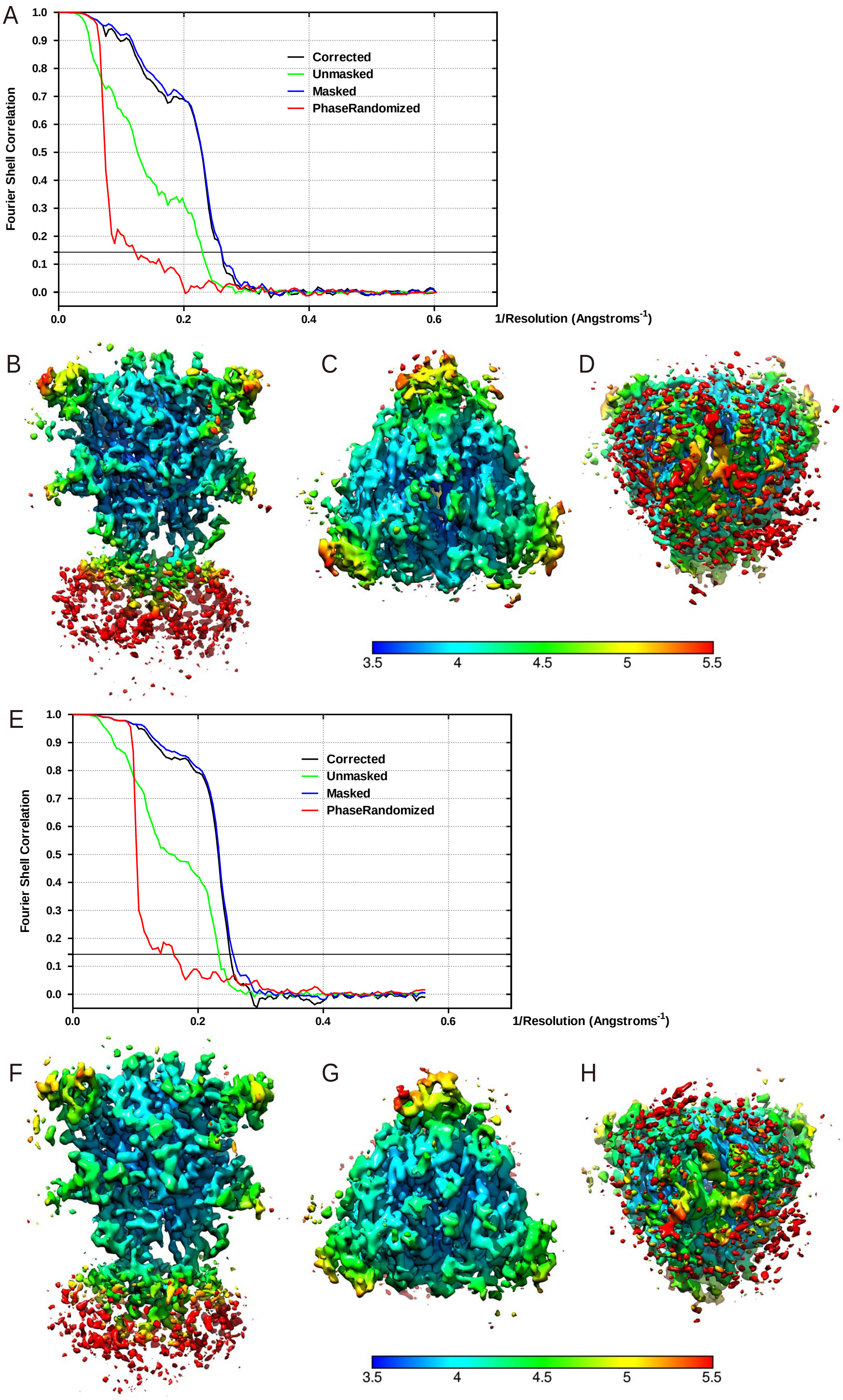

Supplementary Figure 6. Cryo-EM analyses of zebrafish P2X4 with C1 symmetry. Cryo-EM analyses of zebrafish P2X4 in complex with BX430 (**A-D**) and BAY-1797 (**E-H**). The gold-standard Fourier shell correlation (FSC) curves for resolution estimation (**A, E**). Side views (B, F), top views from the extracellular side (C, G), and bottom views from the cytoplasmic side (D, H) of the EM density maps for zebrafish P2X4 in complex with BX430 and BAY-1797, respectively, colored according to the local resolution, estimated using RELION.

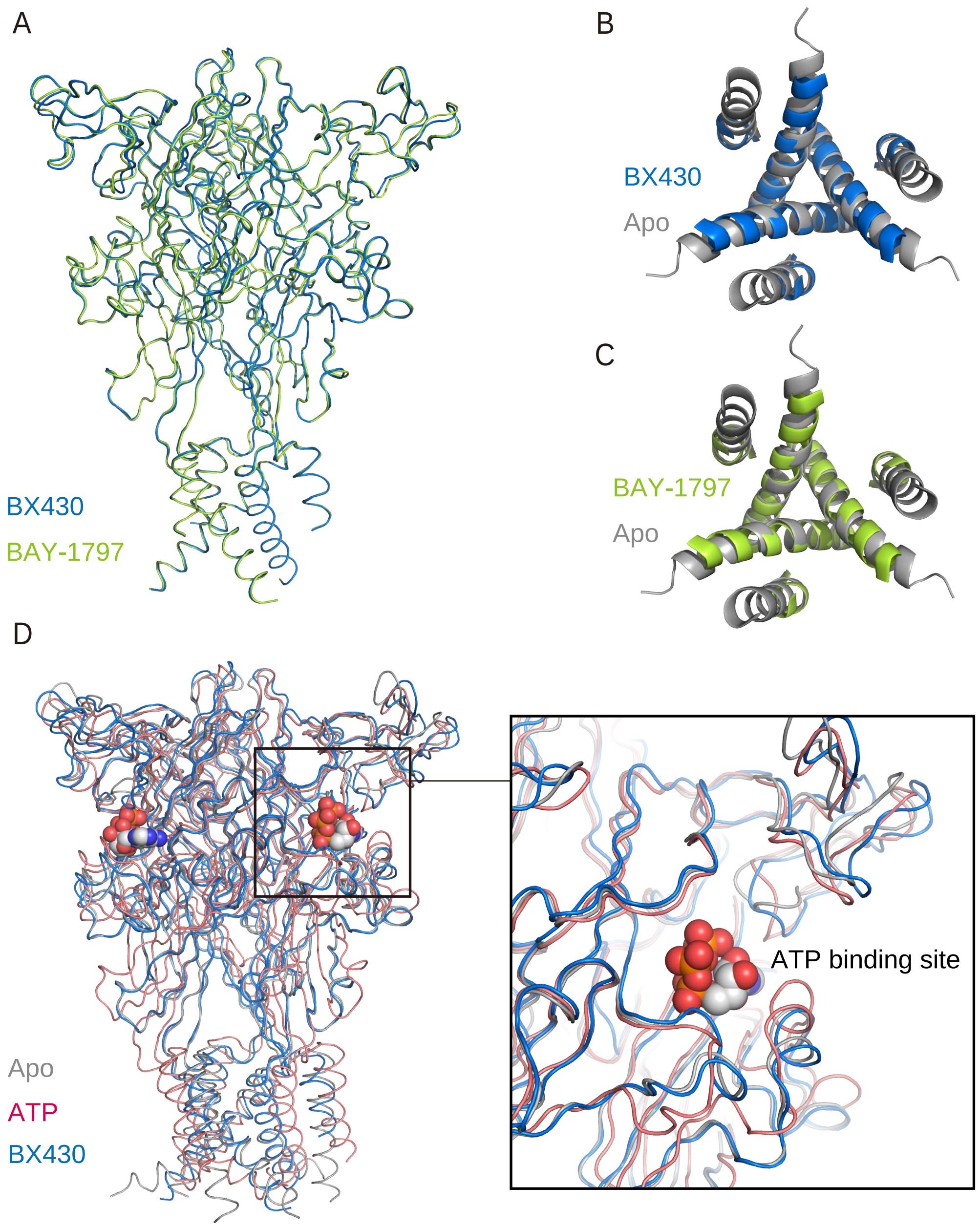

Supplementary Figure 7. Comparison of the BX430-bound and BAY-1797-bound structures.
(**A**) Superimposition of the BX430-bound structure (blue) onto the BAY-1797 structure (green). (**B, C**) Superimposition of the BX430-bound (**B**) and BAY-1797-bound (**C**) structures onto the apo structure (PDB ID: 4DW0, gray). Only the TM helices are shown and viewed from the intracellular side. (**D**) Superimposition of the BX430-bound structure (blue) and ATP-bound structure (PDB ID: 4DW1, red) onto the apo structure (gray). ATP molecules are shown in sphere representation.

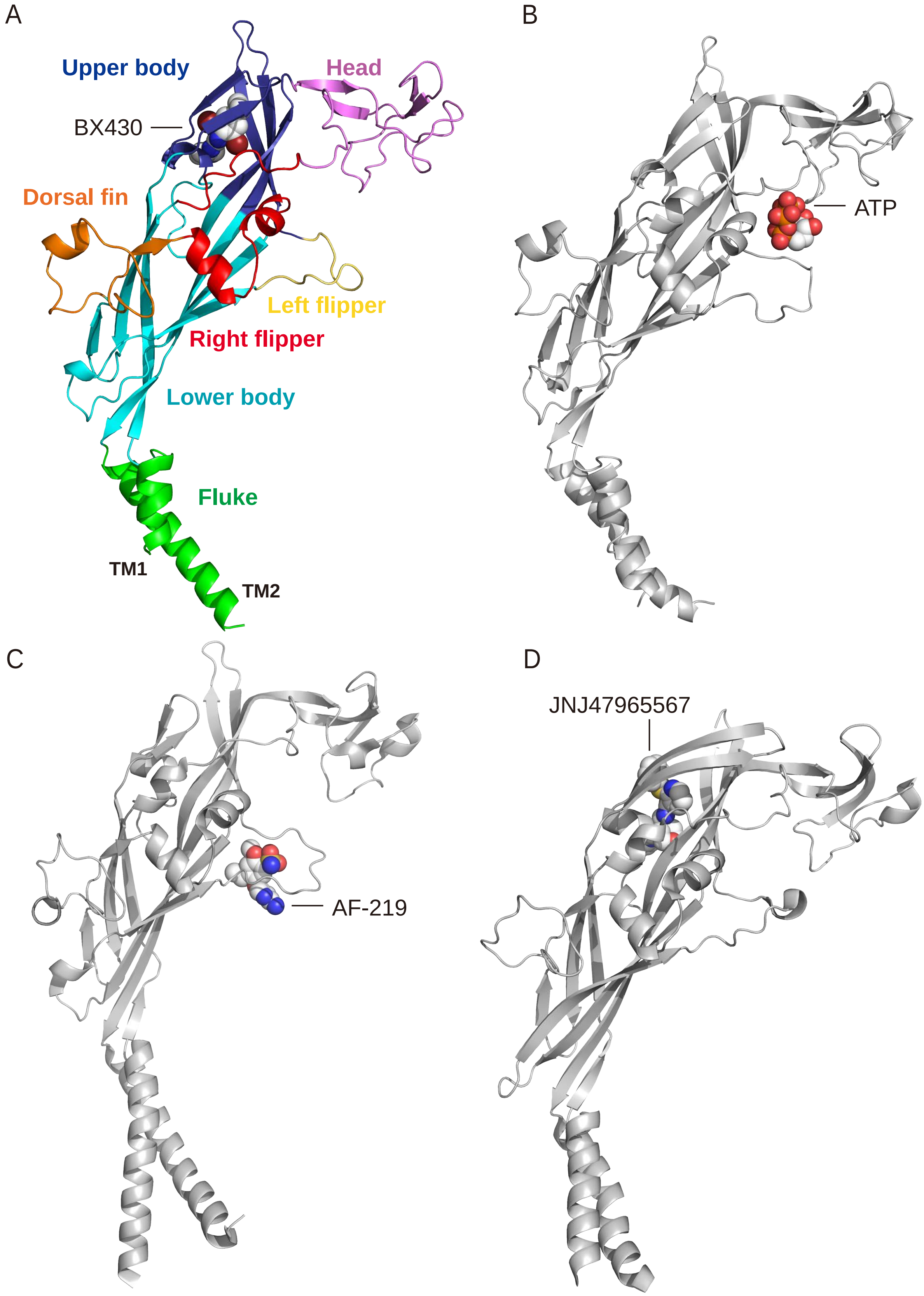

Supplementary Figure 8. Dolphin model of the P2X subunit.
(**A**) Each region of the P2X4 promoter in cartoon representations is colored according to the dolphin model. (**B-D**) Each protomer of the ATP-bound P2X4 structure (**B**) (PDB ID: 4DW1), the AF-219-bound P2X3 structure (**C**) (PDB ID: 5YVE) and the JNJ47965567-bound P2X7 structure (**D**) (PDB ID: 5U1X) is shown in gray.

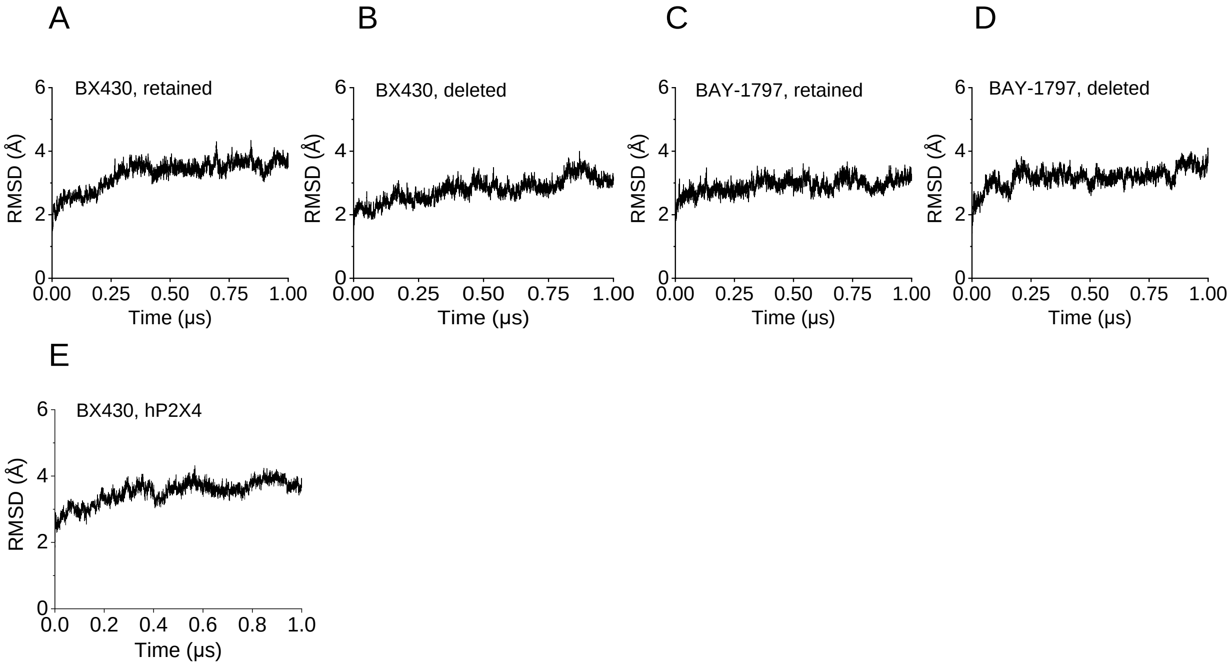

Supplementary Figure 9. Plots of the RMSD values for Cα atoms during the MD simulations

MD simulations using the BX430-bound structure with BX430 retained (A) or deleted (B), the BAY-1797-bound structure with BAY-1797 retained (C) or deleted (D) and the BX430-bound human P2X4 structural model based on the BX430-bound zfP2X4 structure (E) as starting models. The plots of the root mean square deviations (RMSD) for Cα atoms during the MD simulations are shown. Two more additional repeats of the MD simulations were shown in Supplementary Fig. 13A-13H for (A-D) and 14A-14B for (E). In total, MD simulations were performed three times.

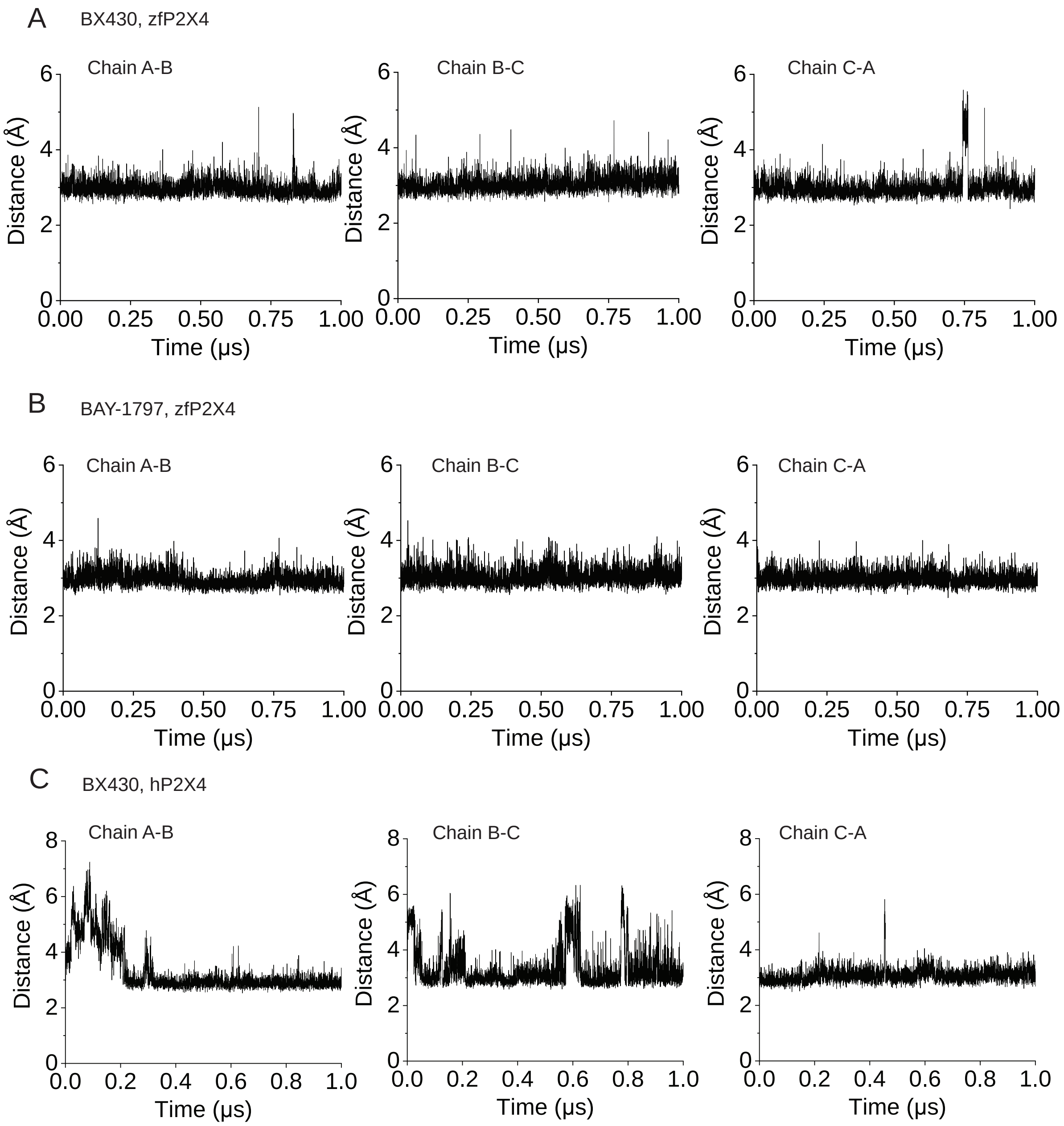

Supplementary Figure 10. Distance plots between the amine groups of BX430 and BAY-1797 and the main chain carbonyl of Asp91(zfP2X4) or Asp88 (hP2X4).
MD simulations of the BX430-bound (A) and of the BAY-1797-bound (B) structure of zfP2X4 and of the BX430-bound structural model of hP2X4 based on the BX430-bound zfP2X4 structure (C). Plots of the distances between the amine group (N9) of BX430 and the main chain carbonyl of Asp91 (zfP2X4) of two adjacent subunits (A) and between the amine group (N10) of BAY-1797 and the main chain carbonyl of Asp91 of two adjacent subunits (B) are shown. Plots of the distances between the amine group (N9) of BX430 and the main chain carbonyl of Asp88 (hP2X4) of two adjacent subunits (C) are shown. Two more additional repeats of the MD simulations were shown in Supplementary Fig. 13I-13L for (A-B) and 14C-14D for (C). In total, MD simulations were performed three times.

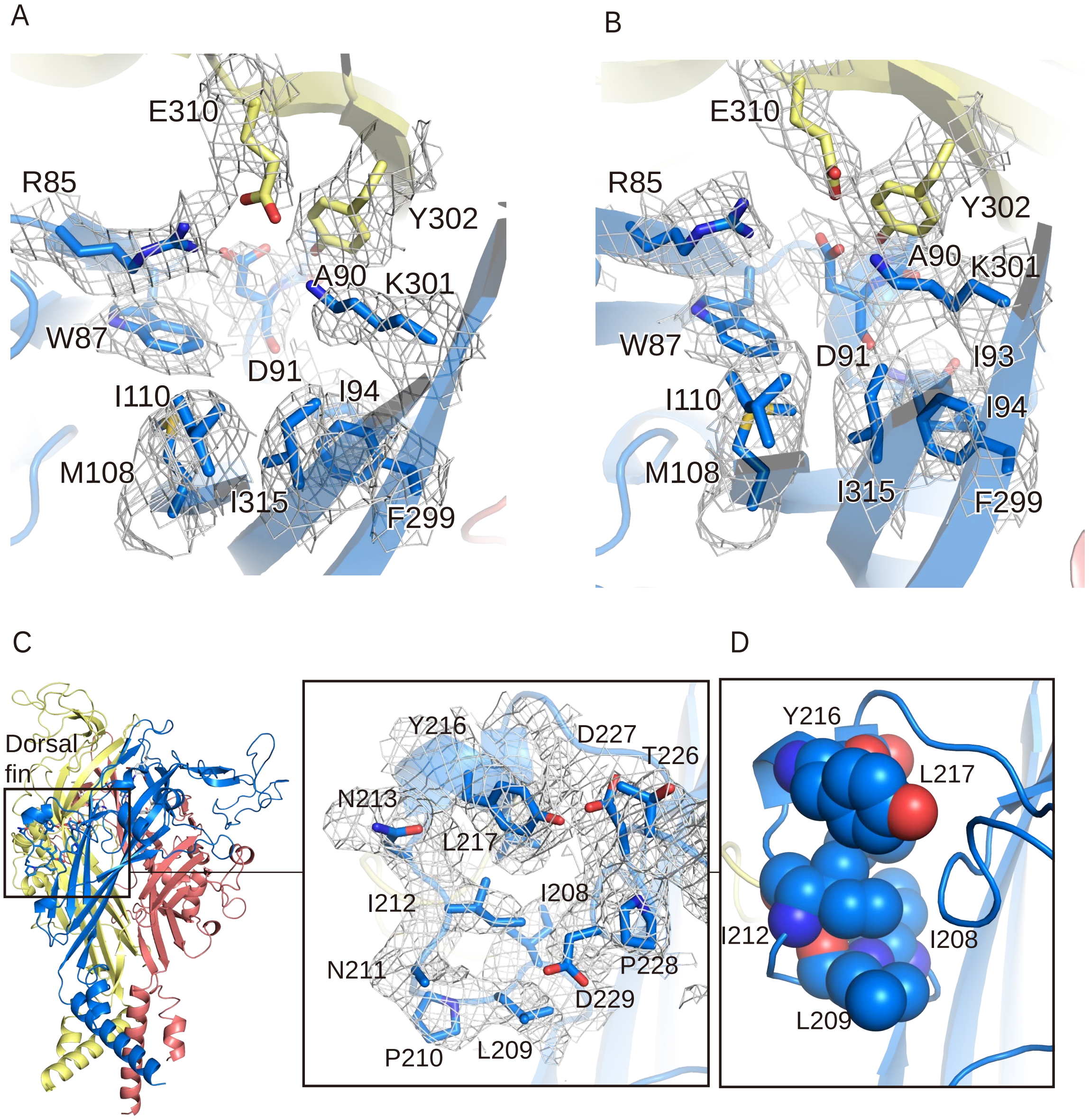

Supplementary Figure 11. EM density maps for binding pockets and the dorsal fin region in the BX430-bound structure.

(**A**) The EM density maps for the residues involved in BX430 interaction are shown and contoured at 4.0 σ. (**B**) The EM density maps for the residues involved in BAY-1797 interaction are shown and contoured at 4.0 σ. (**C**)The EM density map for the dorsal fin region is shown and contoured at 5.0 σ. (**D**) Ile212 and neighboring residues at the dorsal fin region are shown as spheres.

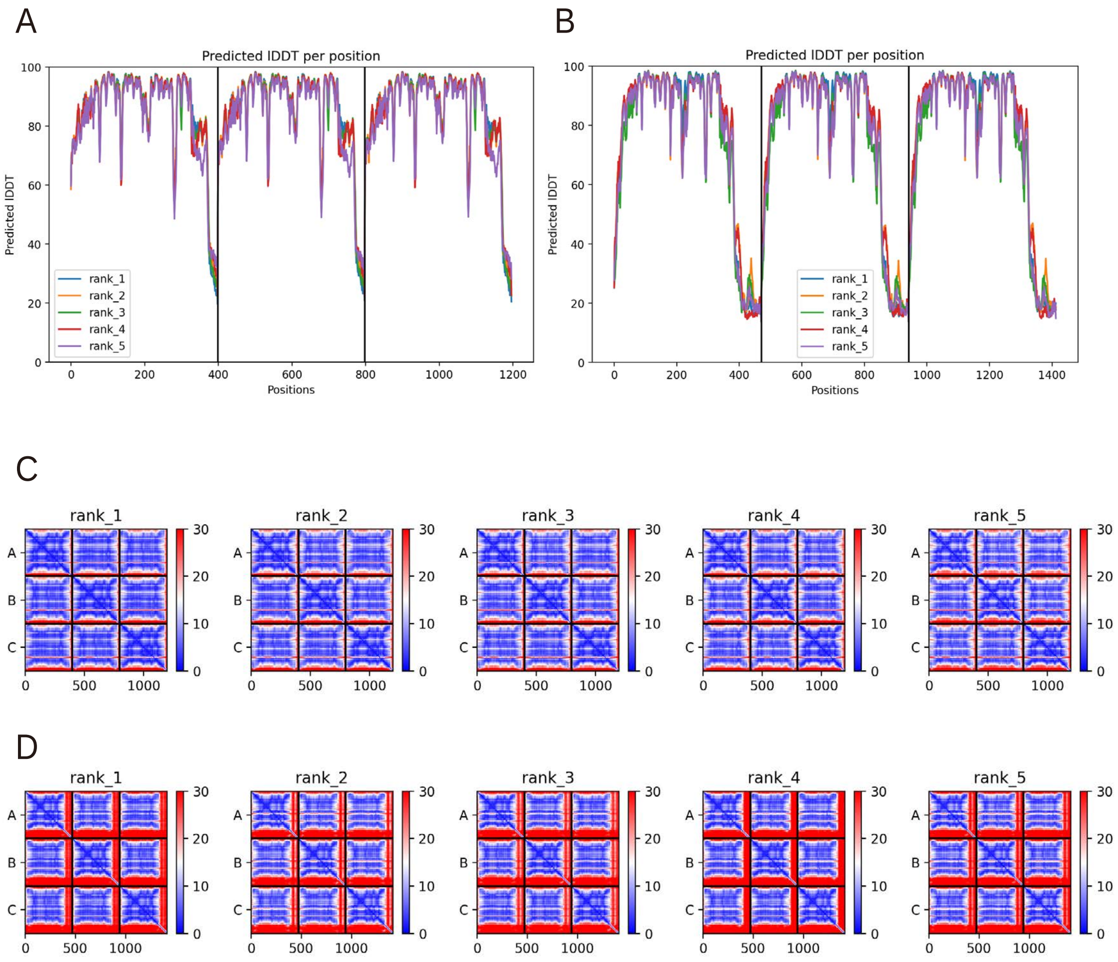

Supplementary Figure 12. AlphaFold prediction statistics
(**A, B**) Predicted local distance difference test (pLDDT) scores for each amino acid position of the hP2X1 trimer (**A**) and hP2X2 trimer (**B**). (**C, D**) Predicted aligned error maps for the hP2X1 trimer (**C**) and hP2X2 trimer (**D**). The top-ranked prediction was used for Fig. 8. These figures were generated automatically by ColabFold.

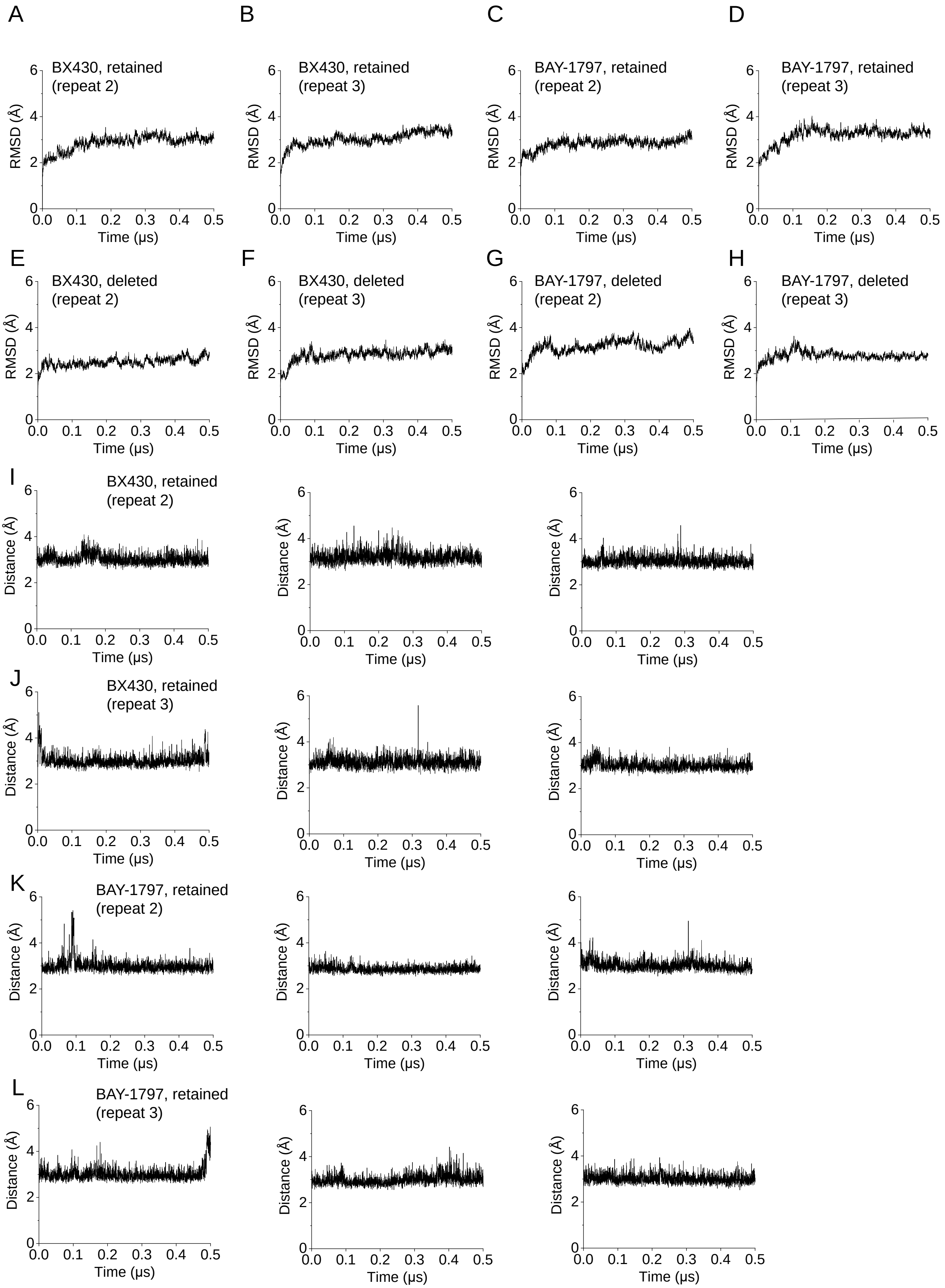

Supplementary Figure 13. Repetitions of MD simulations of zfP2X4 in complex with BX430 and BAY-1797 (Continued)

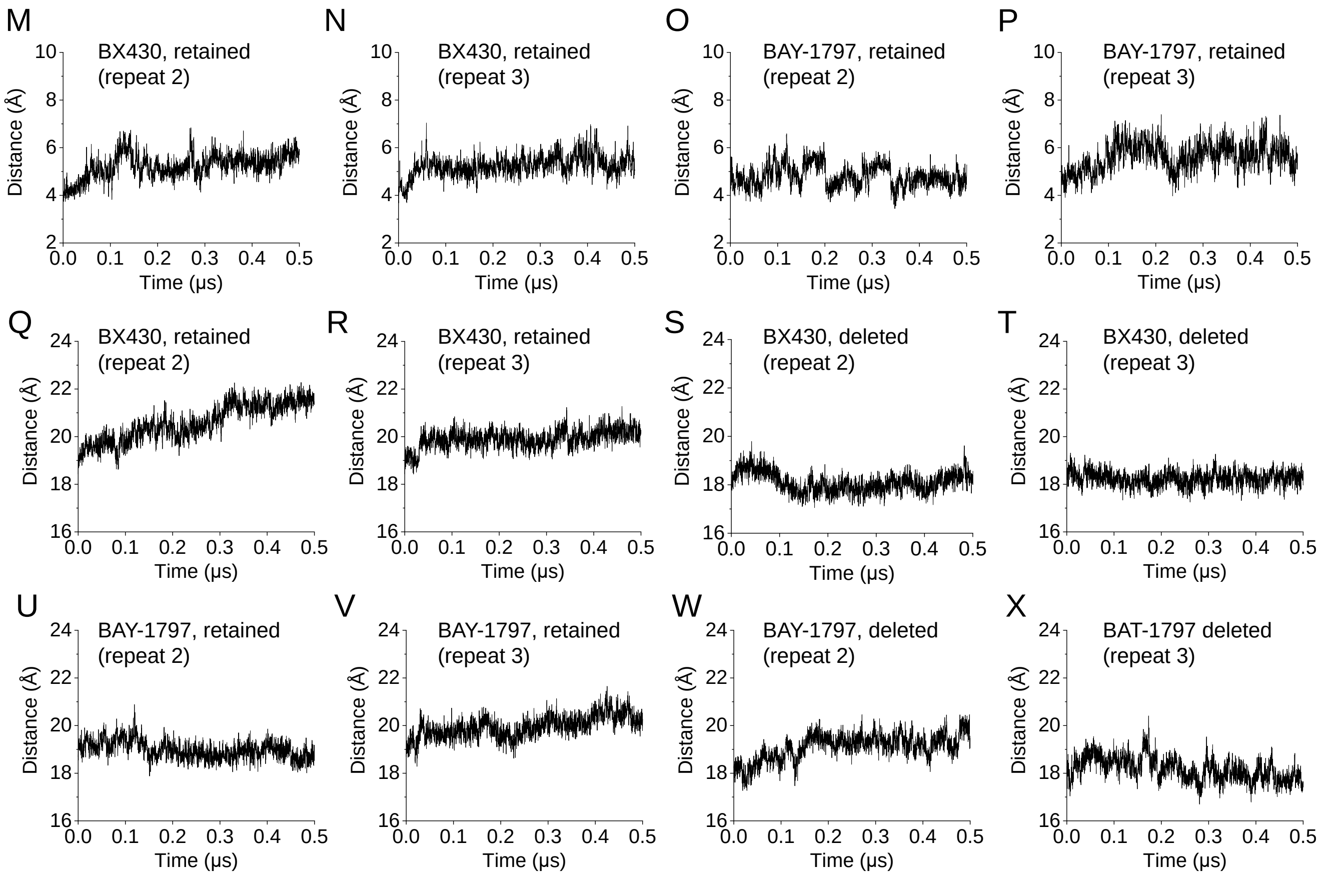

Supplementary Figure 13. Repetitions of MD simulations of zfP2X4 in complex with BX430 and BAY-1797.

(**A-H**) Plots of the root mean square deviations (RMSD) for Cα atoms during the repeated MD simulations using the BX430-bound structure with BX430 retained (**A, B**) or the BAY-1797-bound structure with BAY-1797 retained (**C, D**) and the BX430-bound structure with BX430 deleted (**E, F**) or the BAY-1797-bound structure with BAY-1797 deleted (**G, H**). (**I-L**) Plots of the distances between the amine group (N9) of BX430 and the main chain carbonyl of Asp91 (zfP2X4) of two adjacent subunits (**I, J**) and between the amine group (N10) of BAY-1797 and the main chain carbonyl of Asp91 of two adjacent subunits (**K, L**) are shown. (**M-P**) Plots of the distances between the NZ atom of Lys301 and the center of the aromatic ring of Tyr302 of two adjacent subunits in the BX430-bound structure (**M, N**) and the BAY-1797-bound structure (**O, P**) are shown. (Q-X) The distance plots of Cα atoms between Ile110 and Gln116 of two adjacent subunits in the BX430-bound structure (**Q, R**), the BAY-1797-bound structure (**U, V**), and the BX430-bound structure with BX430 deleted (**S, T**) or the BAY-1797-bound structure with BAY-1797 deleted (**W, X**) are shown.

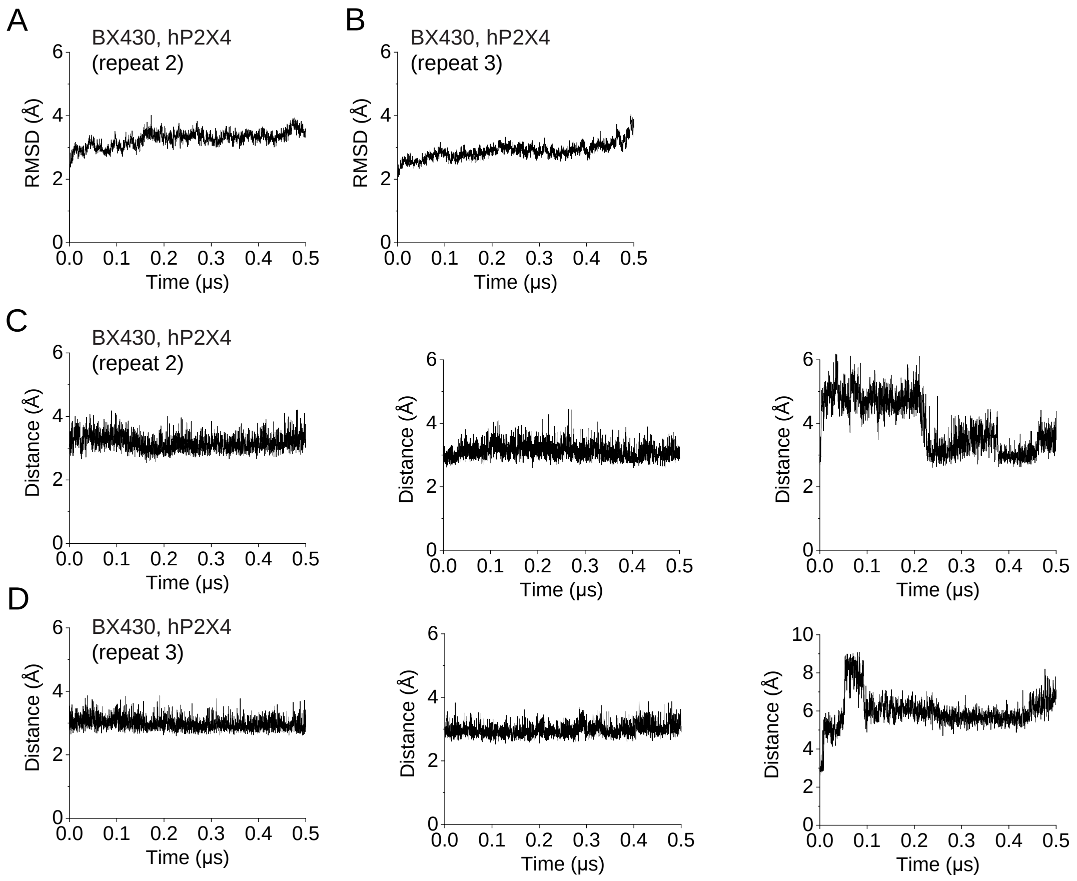

Supplementary Figure 14. Repetitions of MD simulations of the BX430-bound structure of hP2X4 modeled from the BX430-bound zfP2X4 structure.
(**A, B**) Plots of the root mean square deviations (RMSD) for Cα atoms during the repeated MD simulations using the BX430-bound structure of hP2X4 modeled from the BX430-bound zfP2X4 structure. (**C, D**) Plots of the distances between the amine group (N9) of BX430 and the main chain carbonyl of Asp88 (hP2X4) of two adjacent subunits are shown.

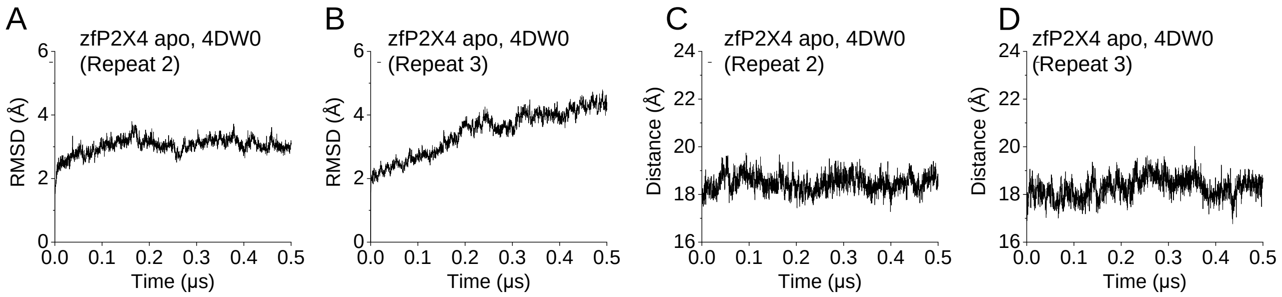

Supplementary Figure 15. Repetitions of MD simulations of the previously published apo structure of zfP2X4 (PDB ID: 4DW0)

The plots of the root mean square deviations (RMSD) for Cα atoms (**A, B**) and distance of Cα atoms between Ile110 and Gln116 of two adjacent subunits (**C, D**) are shown.

Supplementary Table 1. Summary of the system setup in MD simulations.

	Total number of atoms	Total number of water molecules	Box sizes (Å* Å* Å)
zfP2X4-BX430-1	141891	30652	97.9*101.5*138.9
ZfP2X4-BX430-2	137668	28979	97.5*101.1*139.0
ZfP2X4-BX430-3	137668	28979	97.5*101.1*139.0
zfP2X4-BX430-without-BX430-1	141945	30884	97.7*101.4*139.4
zfP2X4-BX430-without-BX430-2	137232	28915	97.3*101.0*139.0
zfP2X4-BX430-without-BX430-3	137232	28915	97.3*101.0*139.0
ZfP2X4-BAY-1	139185	29787	95.3*103.1*137.6
ZfP2X4-BAY-2	134832	28116	94.7*102.4*138.6
ZfP2X4-BAY-3	134832	28116	94.7*102.4*138.6
zfP2X4-BAY-without-BAY-1	145317	31741	100.8*98.7*141.8
zfP2X4-BAY-without-BAY-2	132297	27543	95.0*99.6*139.3
zfP2X4-BAY-without-BAY-3	132297	27543	95.0*99.6*139.3
ZfP2X4-apo-1	129918	27256	99.3*94.7*137.6
ZfP2X4-apo-2	136570	28983	95.9*99.8*141.9
ZfP2X4-apo-3	134523	28614	99.6*95.4*138.1
hP2X4-BX430-1	134287	28614	96.9*98.5*140.7
hP2X4-BX430-2	134287	28614	96.9*98.5*140.7
hP2X4-BX430-3	134917	28512	97.3*98.2*140.6
