# Supplementary material for: Structural insights into the allosteric inhibition of P2X4 receptors
Source: Nat Commun. 2023 Oct 13;14:6437. doi: 10.1038/s41467-023-42164-y (PMC10575874; doi:10.1038/s41467-023-42164-y)
Supplement: Supplementary file 3 — Description of additional supplementary files [file 41467_2023_42164_MOESM3_ESM.pdf]

Description of Additional Supplementary Files Document

Supplementary Dataset 1 - AlphaFold prediction model and log files from ColabFold for human P2X1

Supplementary Dataset 2 - AlphaFold prediction model and log files from ColabFold for human P2X2.

Supplementary Movie 1 - BX430-dependent structural changes of zfP2X4.
