# Supplementary figures and images for: Structural insights into the allosteric inhibition of P2X4 receptors
Source: Nat Commun. 2023 Oct 13;14:6437. doi: 10.1038/s41467-023-42164-y (PMC10575874; doi:10.1038/s41467-023-42164-y)

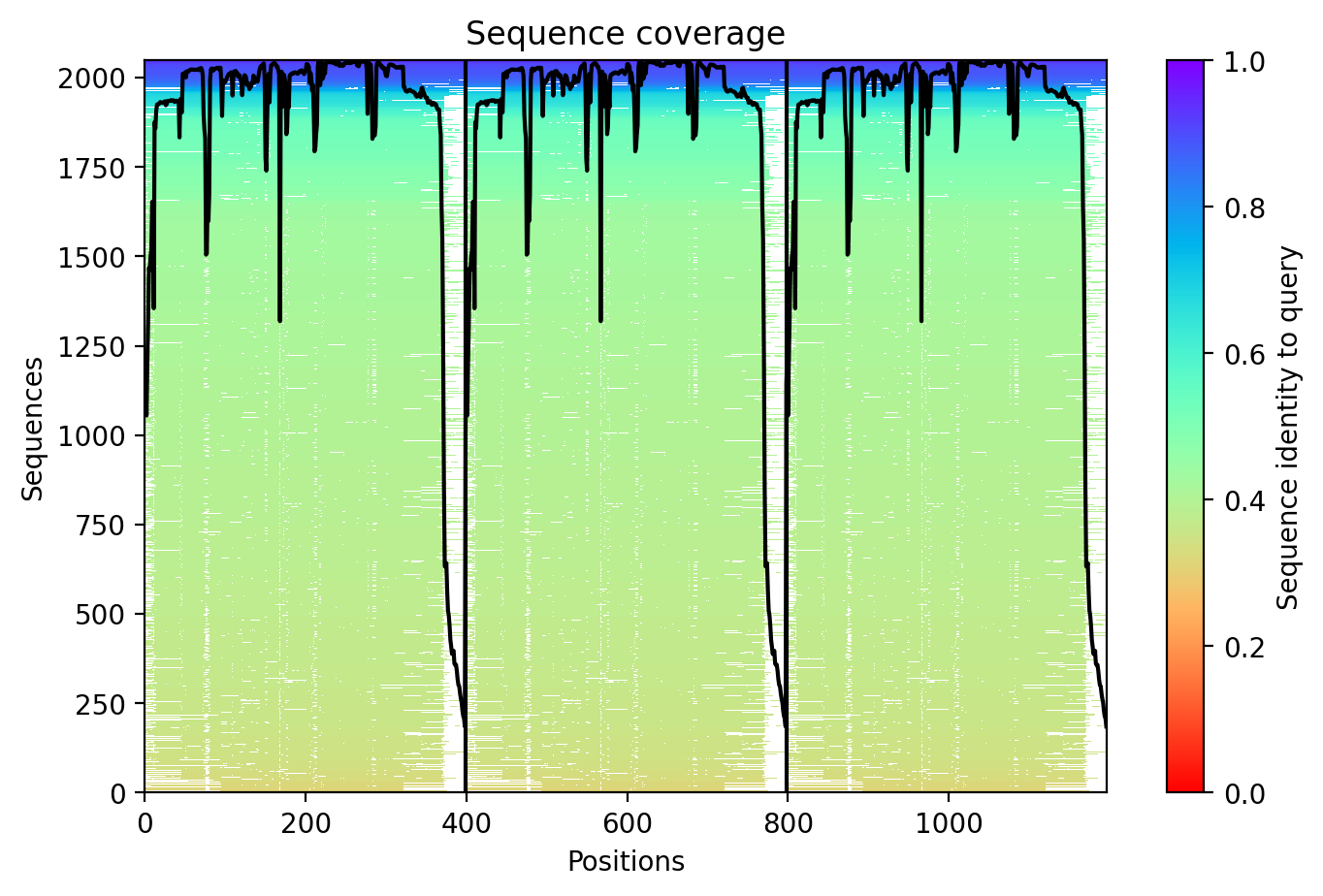

Supplement: Supplementary file 4 — Supplementary Dataset 1 [file 41467_2023_42164_MOESM4_ESM.zip › hP2X1trimer_a111a/hP2X1trimer_a111a_coverage.png]

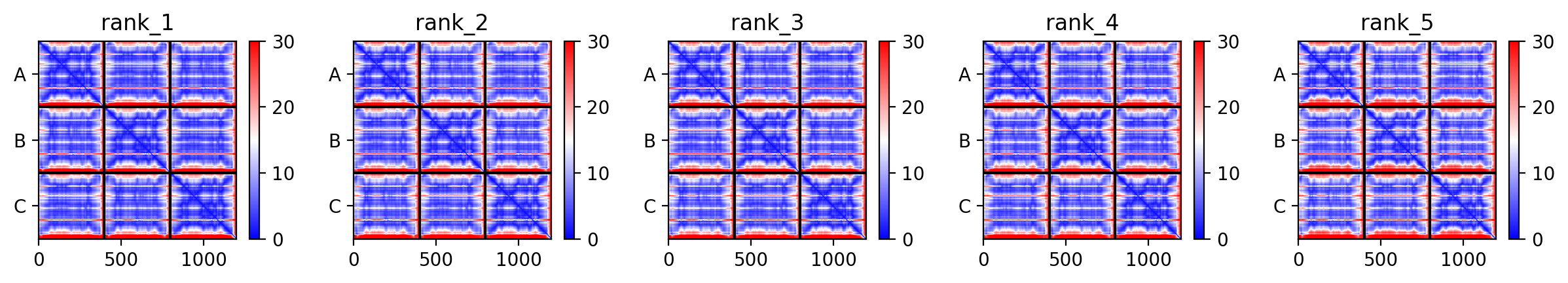

Supplement: Supplementary file 4 — Supplementary Dataset 1 [file 41467_2023_42164_MOESM4_ESM.zip › hP2X1trimer_a111a/hP2X1trimer_a111a_pae.png]

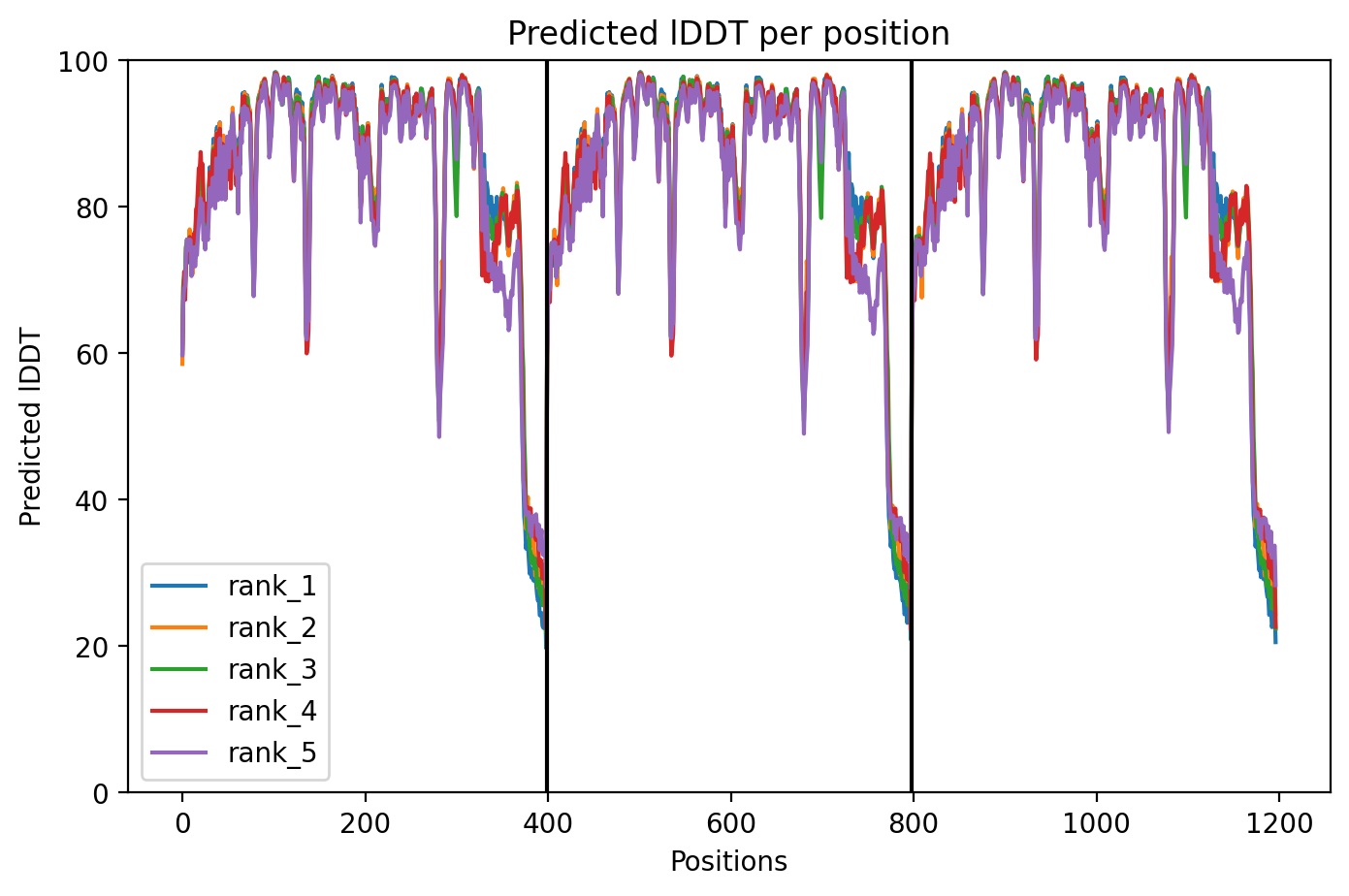

Supplement: Supplementary file 4 — Supplementary Dataset 1 [file 41467_2023_42164_MOESM4_ESM.zip › hP2X1trimer_a111a/hP2X1trimer_a111a_plddt.png]

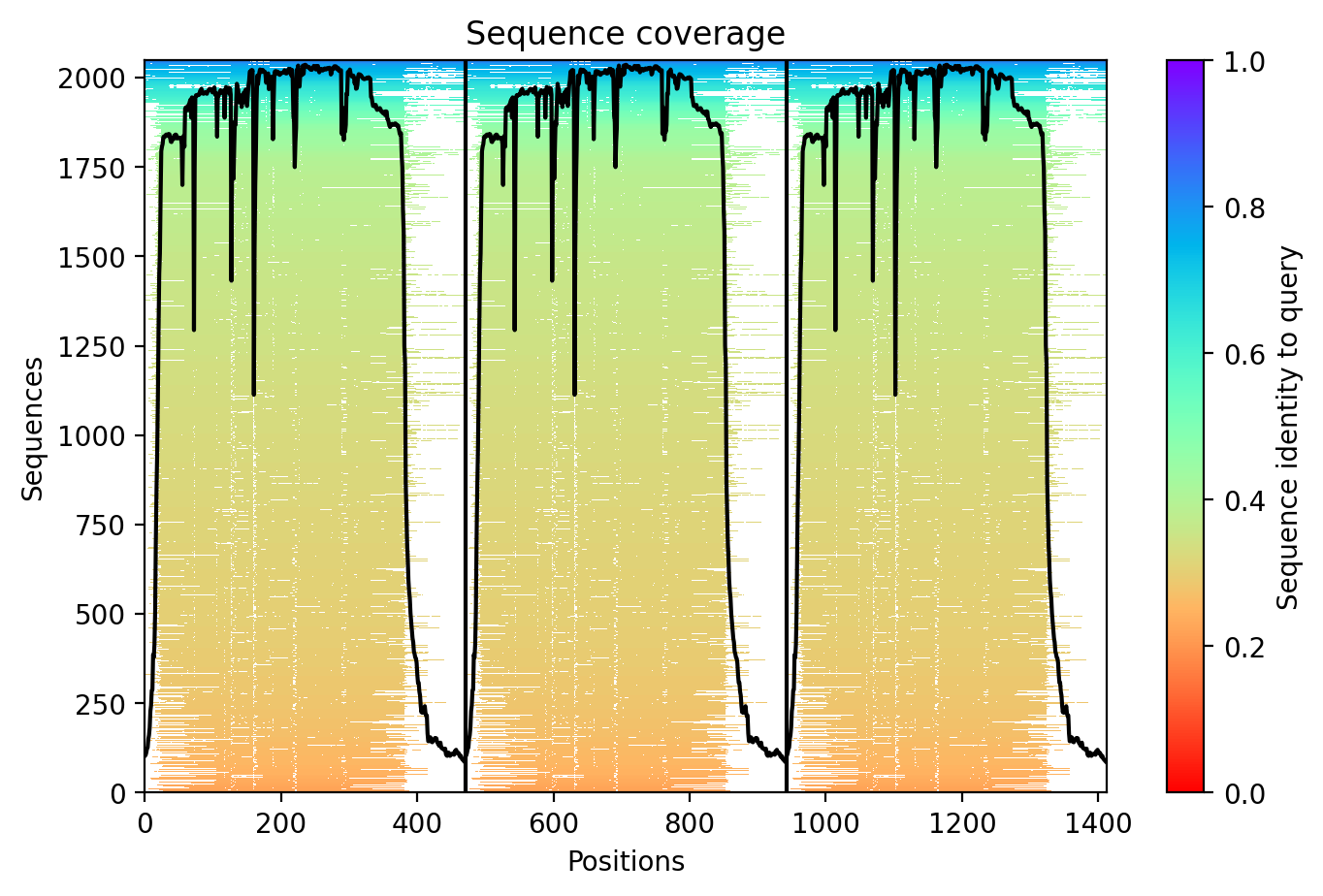

Supplement: Supplementary file 5 — Supplementary Dataset 2 [file 41467_2023_42164_MOESM5_ESM.zip › hP2X2trimer_488a3/hP2X2trimer_488a3_coverage.png]

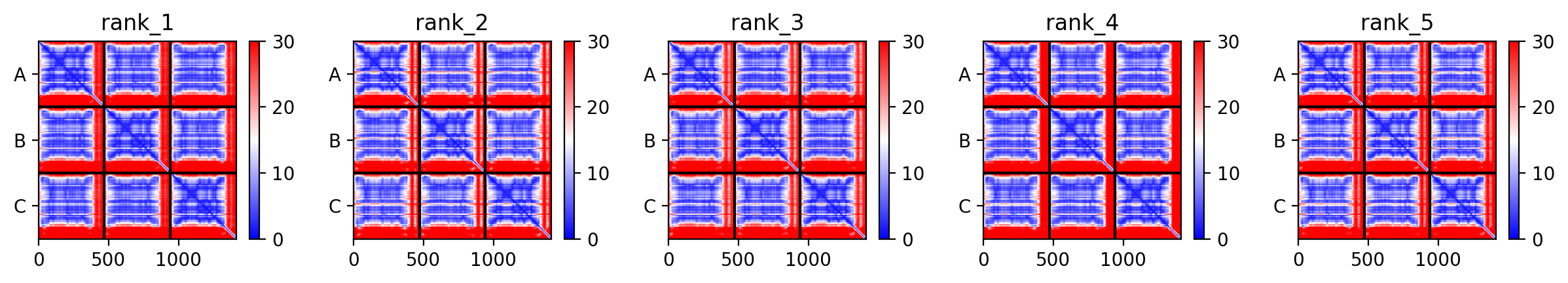

Supplement: Supplementary file 5 — Supplementary Dataset 2 [file 41467_2023_42164_MOESM5_ESM.zip › hP2X2trimer_488a3/hP2X2trimer_488a3_pae.png]

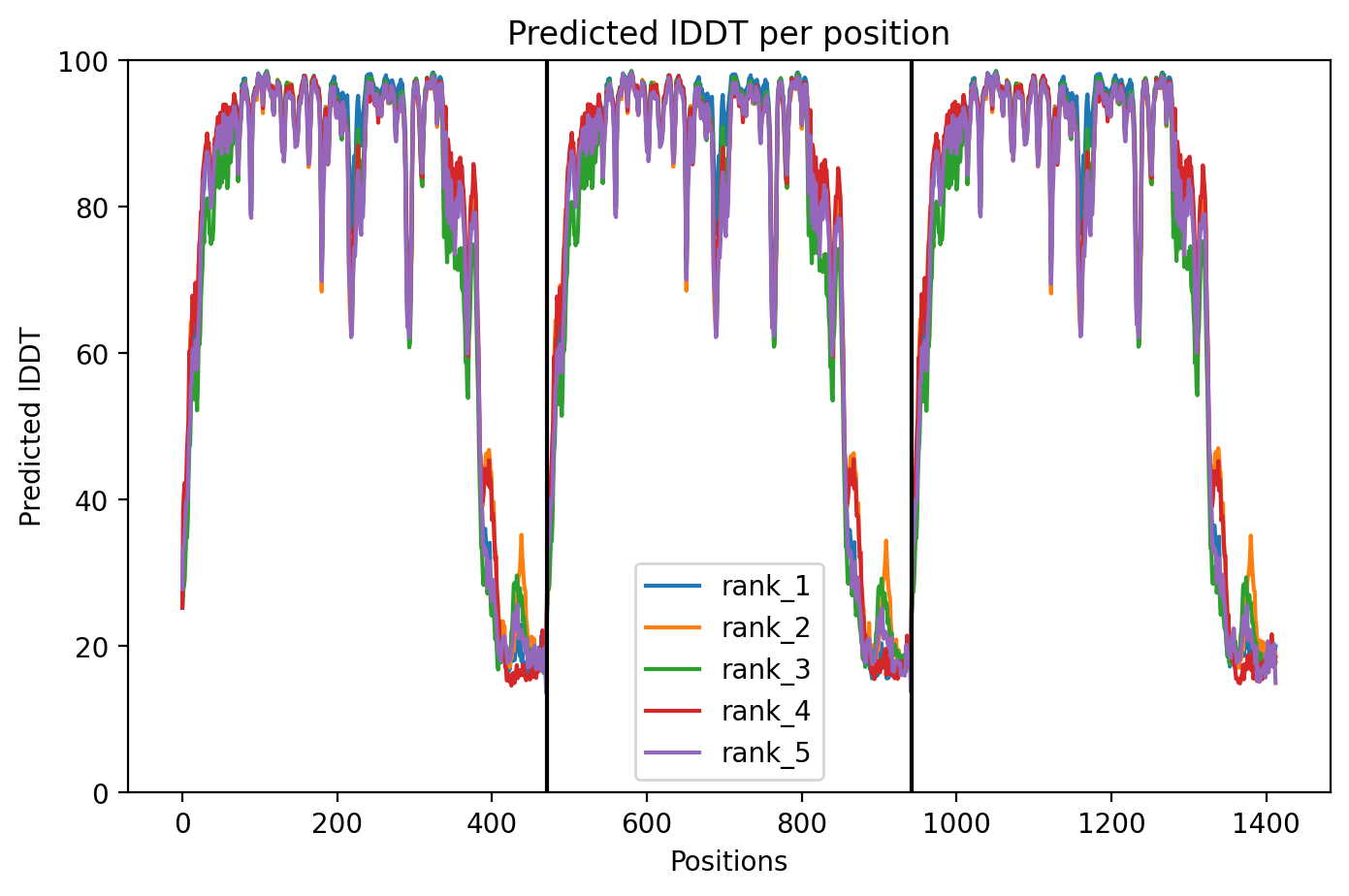

Supplement: Supplementary file 5 — Supplementary Dataset 2 [file 41467_2023_42164_MOESM5_ESM.zip › hP2X2trimer_488a3/hP2X2trimer_488a3_plddt.png]
